# ICU nurses' perceptions of patients with co‐morbid mental health disorders: An integrative review

**DOI:** 10.1111/nicc.70022

**Published:** 2025-03-31

**Authors:** Angela Teece, John Baker

**Affiliations:** ^1^ Lecturer in Adult Nursing University of Leeds Leeds UK; ^2^ Chair of Mental Health Nursing University of Leeds Leeds UK

**Keywords:** bias and stigma, ICU nursing, mental health

## Abstract

**Background:**

The prevalence of patients with a mental health (MH) disorder in intensive care units (ICU) is roughly twice that of other secondary care areas. This patient group can struggle to access the health care system because of stigma. Nurses' perceptions of MH patients in the Emergency Department have been studied and were associated with avoidance, misconceptions and perceived lack of skills to manage this patient group; however, it was unclear if similar issues were present amongst ICU nurses.

**Aim:**

This review aimed to explore how nurses perceive ICU patients with a co‐morbid MH disorder.

**Study Design:**

An integrative review was undertaken in March 2024 using CINAHL, Medline, Embase and PsychInfo to synthesize empirical and theoretical evidence from a range of different research approaches. A five‐step approach (problem identification, literature search, data evaluation using the Mixed Methods Appraisal tool, data analysis and presentation) was followed. Papers were included if they focused on nurses' perceptions of adult ICU patients with a co‐morbid MH disorder. Totally, 620 studies were identified following duplicate removal.

**Results:**

Eight studies were selected for inclusion. Four themes were identified: (1) ‘Those types of patient’, (2) Patients with MH disorders are all violent and aggressive, (3) ‘They’ don't belong in ICU and (4) ‘They’ need someone with special skills. The themes explored issues of preconceptions, stigma and ‘othering’.

**Conclusions:**

There was a paucity of research on this topic, and it was limited in geographical area. The findings suggest that stigma, misconceptions, a lack of support and a perceived lack of skills might lead nurses to deliver suboptimal care to this vulnerable patient group.

**Relevance to Clinical Practice:**

Stigma against patients with MH disorders could lead ICU nurses to reduce their engagement with them, impacting negatively on the provision of holistic care. Education and ongoing support are required to reduce misconceptions and bias and increase nurses' confidence when managing patients with co‐morbid MH disorders.

**What is known about the topic**

- Patients with mental health disorders can struggle to access health care.
- The prevalence of mental health disorders in intensive care unit (ICU) patients is twice that of patients in other areas of secondary care.
- Nurses are susceptible to socially generated biases and stigma. Unconsciously or consciously acting on these views can lead to a decrease in the quality of holistic care.

**What this paper adds**

- There is a paucity of high‐quality research in this area of practice. The studies included in this review suggest that nurses may deliver care influenced by socially generated biases and stigma.
- Assumptions about mental health patients being violent and aggressive can lead to increased use of restrictive practices and staff being unwilling to engage.
- ICU nurses appear to lack confidence in their skills when managing patients with mental health disorders. A need for greater support and education has been identified.

## 1 INTRODUCTION

Approximately 25% of people will experience some kind of mental health (MH) disorder during their lifetime.^1^ The presence of a mental illness is known to exacerbate the risk of physical illness, and people with a serious mental illness (SMI) diagnosis experience a mortality rate of up to four times that of the rest of the population.^2^, ^3^ Patients with a MH disorder, such as schizophrenia or major depression, are disproportionately overrepresented in the intensive care population,^4^ with a prevalence of roughly twice that of other patient populations in secondary care.

Intensive care units (ICUs) are a specialized area of secondary care, where patients requiring single or multi‐organ support can be cared for with a high level of medical and nursing supervision.^5^ Patients with MH disorders form a unique subset of the intensive care population, requiring additional psychological support from bedside nurses^4^ and being at a greater risk of a prolonged stay^4^ and the development of delirium.^6^ It is therefore important that critical care nurses consider the unique needs of this subset of patients.

### 1.1 Background

Stigma is a socially generated negative attitude about a person or group.^7^ Stigma against patients with MH disorders has led to their devaluation and perceived exclusion from some health care services.^7^, ^8^, ^9^ Nurses are bound by their professional code to deliver unbiased, high‐quality care^10^; however, they remain susceptible to personal and socially disseminated views about mental illness. The topic of stigma against MH disorders has been studied amongst nurses who work in the emergency department (ED).^11^, ^12^ Participants expressed concerns about the appropriateness of the ED environment for this patient group, about inadequacy regarding their own skills, and the impact of negative perceptions that they hold towards mental illness. The disempowerment and application of stigma to patients with MH disorders is also prevalent in secondary and intensive care^2^, ^3^ and can have a detrimental impact on the provision of holistic care.^13^ The high prevalence of patients with a MH disorder in ICU^4^ alongside the vulnerability of critically ill patients and the position of power occupied by the ICU nurse^14^ makes these preconceptions and their possible impact on care provision an important topic to explore.

### 1.2 Aim and objectives

This review aimed to explore how nurses perceive ICU patients with a co‐morbid MH disorder through identifying and analysing appropriate literature and synthesizing evidence from a range of research designs.^15^ This review will inform future empirical research in this area.

## 2 METHODS

### 2.1 Design

An integrative review was undertaken. This type of review facilitates the synthesis of empirical and theoretical evidence from a range of different research approaches^16^ and was deemed an appropriate approach for a review that focused on an under‐researched topic and therefore needed to capture diverse methodological approaches.

Whittemore and Knafl^16^ outlined a five‐step approach (problem identification, literature search, data evaluation, data analysis and presentation). The identified problem was the apparent paucity of knowledge regarding how ICU nurses perceive patients under their care who have a co‐morbid MH disorder.

### 2.2 Search strategy

A comprehensive search of electronic databases (CINAHL, Medline, Embase and PsychInfo) was undertaken in March 2024 using four facets (setting, nurses, attitudes and MH disorders) (Table 1). Keywords within each facet were combined with the Boolean operator ‘OR’, and then facets were combined using ‘AND’. The search was limited to English language papers from 2005 onwards to capture contemporary views on MH.

**TABLE 1:** Search facets and keywords.

Facet 1: Setting	Facet 2: Attitudes	Facet 3: Mental Health	Facet 4: Nurses
ICU	Stereotype	Mental health	Nurse
HDU	Stigma	Mental illness	Nursing
ITU	Prejudice	Mental disorder	
Intensive care	Judgement	Psychosis/psych*	
High dependency	Perception	Suicide	
Critical care	Attitude	Self‐harm	
	Opinion	Anxiety & depression	
	Generalization	Psychiatric disorder	
	Belief	Psychiatric Illness	

Abbreviation: HDU, High dependency unit; ICU, intensive care unit; ITU, intensive therapy unit.

**FIGURE 1:**
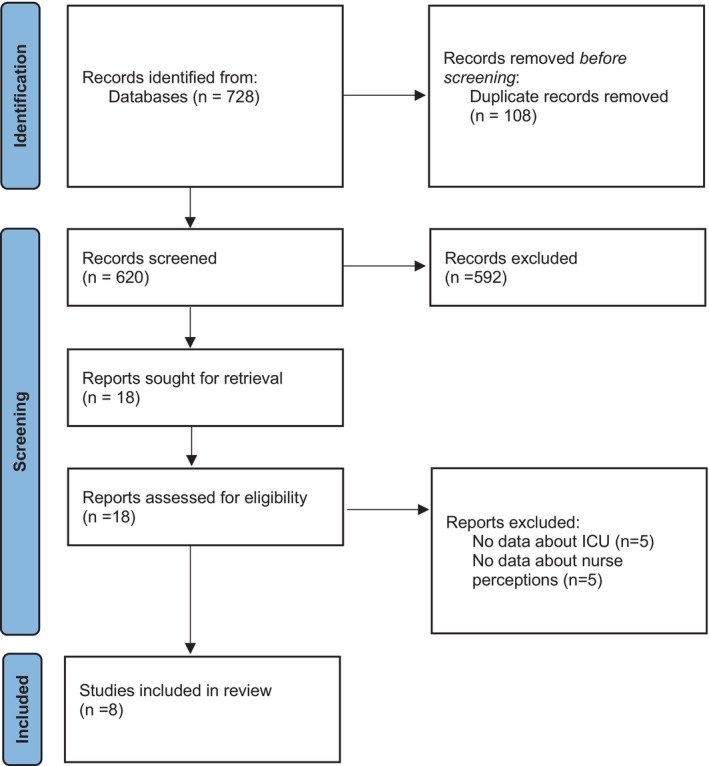
PRISMA diagram.^17^

A total of 728 papers were returned (CINHAL: 483, Medline: 123, Embase: 92, PsychInfo: 26). Following the removal of duplicates (*n* = 108), title, abstract and then full‐text screening were undertaken (AT). The bulk of the studies were excluded at title screening as they focused on nurses' perceptions of their own MH, rather than that of their patients. Studies set in non‐ICU environments were also excluded as non‐pertinent to the focus of this review (Figure 1). The review included studies worldwide. Papers were included if they included nurses' perceptions of adult ICU patients with clear co‐morbid MH disorders. All types of MH disorder were included (Table 1). Handsearching of the included papers' reference lists was undertaken. No further results were yielded. The included papers were agreed upon by both authors. The review process followed the PRISMA reporting guidelines.^17^

### 2.3 Data synthesis and evaluation

A convergent data‐based synthesis was then undertaken.^18^, ^19^ This required the transformation of extracted data to enable a qualitative synthesis of the mixed study types included in this review.^20^ Whittemore and Knafl^16^ suggest a process of data reduction. A matrix (Table 2) was created to facilitate the comparison and synthesis of the included studies.

**TABLE 2:** Summary of included studies.

Citation	Country	Objective	Population	Data collection	Analysis	Results	MMAT appraisal	Themes
Aktas & Arabaci (2023)	Turkey	To evaluate of the views of intensive care nurses on the psychological care needs of patients	Fifteen ICU nurses	Individual semi‐structured interviews	Content analysis	ICU nurses try to communicate effectively with patients, but this is made difficult if they have MH disorders. MH disorders are someone else's responsibility to manage. It is not a nursing job. ICU nurses need support to deliver effective care to patients with MH needs.	1.1. Is the qualitative approach appropriate to answer the research question? Yes. 1.2. Are the qualitative data collection methods adequate to address the research question? Yes 1.3. Are the findings adequately derived from the data? Yes, supported with quotations. 1.4. Is the interpretation of results sufficiently substantiated by data? Yes 1.5. Is there coherence between qualitative data sources, collection, analysis and interpretation? Yes	MH patients are violent and aggressive. ‘They’ need someone with special skills.
Bone & Smith (2012)	USA	To explore a case study of a patient with known Bipolar Disorder and ICU delirium and the nursing care and assessments delivered	One male ICU patient	Case study	N/A—critical discussion of a case study	Agitation was associated by the nurse with MH and no delirium assessment was undertaken. ICU recognizes MH diagnoses and then ‘boxes’ them away, prioritising physical health. MH illnesses are considered a separate component rather than a continuum of the patient's health. ICU nurses are uncomfortable with the management of MH illnesses. They can be considered not an ICU responsibility.	1.1. Is the qualitative approach appropriate to answer the research question? Yes 1.2. Are the qualitative data collection methods adequate to address the research question? No formal data collection, but the chosen case study is appropriate. 1.3. Are the findings adequately derived from the data? No formal findings. 1.4. Is the interpretation of results sufficiently substantiated by data? Yes, linked back to the case study. 1.5. Is there coherence between qualitative data sources, collection, analysis and interpretation? Not sure.	‘They’ need someone with special skills
Corfee et al. (2019)	Australia	To explore the ways in which power relationships and the persistent disenfranchisement of consumer/survivors contributed to the reproduction of difference. Explore the social processes around knowledge, power and understanding in ICU	Seventeen female nurses from 8 Australian ICUs	Semi‐structured interviews (up to 1 h)	Constant comparative analysis	Patients with MH diagnoses are seen as inherently dangerous and require greater supervision. Policing of the patient's space. Lower threshold for reacting to distress. Attributed greater potential for violence. Authoritative power is legitimized in ICU. Lack of accommodation and flexibility in care. Patients who are repeatedly admitted are offered poorer care. Patients with MH disorders are seen as dangerous, blame‐worthy and untrustworthy. Othering. Dehumanization (esp DsH). Immediate application of stigma, alternatives not considered.	1.1. Is the qualitative approach appropriate to answer the research question? Yes 1.2. Are the qualitative data collection methods adequate to address the research question? Yes 1.3. Are the findings adequately derived from the data? Yes with added reference to relevant theories. 1.4. Is the interpretation of results sufficiently substantiated by data? Yes, direct quotations included and thorough description of the 1.5. Is there coherence between qualitative data sources, collection, analysis and interpretation? Yes.	MH patients are violent and aggressive. ‘They’ don't belong in ICU. ‘Those types of patients’.
Corfee et al. (2020)	Australia	To explore ways in which the social process of space forms part of a relational arrangement of power and knowledge in intensive care. We argue that space is an integral ingredient that shaped encounters between patients experiencing mental illness and intensive care nurses.	Seventeen female nurses from 8 Australian ICUs	Semi‐structured interviews (up to 1 h)	Iterative cycling between participants' responses, the literature and the theoretical framework	Patients experiencing mental illness were interpreted as a disruption to the ‘business’ of intensive care. The co‐production and reproduction of knowledge and meaning, and the effect of relational aspects of space being pre‐structured by ‘knowledge’ (the symbolic universe of medicine) over time by preceding generations of staff. Those who own (or are the dominant occupants of) the material space have the power to manipulate and organize activities within that space. ICU nurses use space to control and contain. Patients with MH disorders are ‘othered’ and homogenized. ICU is like jail. Power imbalances prevent compassion and empathetic care. Distancing and avoidance. Lack of trust.	1.1. Is the qualitative approach appropriate to answer the research question? Yes 1.2. Are the qualitative data collection methods adequate to address the research question? Yes 1.3. Are the findings adequately derived from the data? Yes with added reference to relevant theories. 1.4. Is the interpretation of results sufficiently substantiated by data? Yes, direct quotations included and thorough description of the 1.5. Is there coherence between qualitative data sources, collection, analysis and interpretation? Yes.	MH patients are violent and aggressive. ‘They’ don't belong in ICU. ‘Those types of patients’. ‘They’ need someone with special skills.
Flaws et al. (2023)	Australia	To address the need for additional education in the management of mental illness in the critical care setting by providing a broad overview of the interrelationship between critical illness and mental illness	Two hypothetical case scenarios	N/A	A discursive paper, drawing on clinical experience and research of the authors and current literature	Patients with mental illness may automatically use coping strategies which have been helpful in other settings but are less effective and potentially counterproductive in ICU, such as expressing discomfort or fear through anger and intimidation. Disorganized behaviour (contrast sedated patients). Illogical behaviour. Staff may judge the ways patients live their lives and experience disgust or anger, leading to guilt and moral distress (burnout). Open communication and validation.	1.1. Is the qualitative approach appropriate to answer the research question? Yes 1.2. Are the qualitative data collection methods adequate to address the research question? No data collection – draws on the authors' experience. 1.3. Are the findings adequately derived from the data? Yes the findings are linked to the case studies. 1.4. Is the interpretation of results sufficiently substantiated by data? No real data. 1.5. Is there coherence between qualitative data sources, collection, analysis and interpretation? Not sure.	MH patients are violent and aggressive. ‘They’ don't belong in ICU. ‘Those types of patients’. ‘They’ need someone with special skills.
Murch (2016)	New Zealand	To explore critical care nurses' attitudes towards caring for patients with mental health issues	Single centre. 30 ICU nurses.	Quantitative survey	Descriptive statistics	Staff had concerns about their personal safety when caring for mental health patients. A majority of the nurses felt confident engaging mental health patients in general conversation. Environment can be a barrier to therapeutic conversation. 37% unsympathetic to patients with multiple admissions.	4.1. Is the sampling strategy relevant to address the research question? Yes. Small single centre study. 4.2. Is the sample representative of the target population? Yes, ICU nurses. But note single centre study. 4.3. Are the measurements appropriate? Yes, descriptive statistics reported. 4.4. Is the risk of non‐response bias low? Non‐response rate not disclosed. 4.5. Is the statistical analysis appropriate to answer the research question? Yes, descriptive statistics.	MH patients are violent and aggressive. ‘They’ don't belong in ICU. ‘Those types of patients’. ‘They’ need someone with special skills.
Patterson et al. (2023)	Australia	To describe and reflect upon aggression towards staff in the intensive care unit (ICU) from the perspectives of staff members	NineteenICU nurses	Semi‐structured interviews	Framework analysis	Patients with a MH diagnosis were seen as especially dangerous and likely to ‘act out’. ‘the self‐harmers’, ‘the suicide attempts’—labelling. These patients cause problems ‘all the time’. Disrupt routine. Negative impact on the recovery of ‘normal’ patients. Not personally or professionally rewarding to care for. Aware of stigmatization but find it hard not to ‘roll your eyes’ at MH patients. MH patients were deemed culpable for any violence and aggression – considered normal for them. Some behaviour attributable to poor management by nursing staff. Most participants shared the interlinked views that mental health and intensive care were ‘at opposite ends of the spectrum’ and required different attitudes to care and required different knowledge and skill sets. Intensive care was chosen by ‘those who don't want to talk deeper, focus on physical cares.’ (Interview 4).	1.1. Is the qualitative approach appropriate to answer the research question? Yes 1.2. Are the qualitative data collection methods adequate to address the research question? Yes 1.3. Are the findings adequately derived from the data? Yes, direct quotations included. 1.4. Is the interpretation of results sufficiently substantiated by data? Yes 1.5. Is there coherence between qualitative data sources, collection, analysis and interpretation? Yes	MH patients are violent and aggressive. ‘They’ don't belong in ICU. ‘Those types of patients’. ‘They’ need someone with special skills.
Weare et al. (2019)	Australia	To examine the knowledge, skills and attitudes of a cohort of Australian nurses towards caring for patients with mental illness in the intensive care unit	Forty ICU nurses	Survey (adapted from various validated tools)	Descriptive statistics	Only 17.5% felt adequately trained to care for patients with mental illness (*n* = 7/40). Some misconceptions were also evident, with a majority of respondents believing that patients with mental illness in ICU were most commonly admitted because of self‐harm (55.0%, *n* = 22/40); and that patients with mental illness were more prone to violence or aggression (67.5%, *n* = 27/40). Concern about ‘saying the wrong thing’. Respondents reported feeling empathy towards patients with mental illness (68.4%, *n* = 26/38). 51.3% (*n* = 19/37) did not believe that patients in ICU received adequate psychological support, and 29.7% (*n* = 11/37) felt that patients with mental illness were not treated with empathy in this unit. Difficulty in understanding self‐harm: such patients are ‘a waste of their time’ (8.1%, *n* = 3/37) or that individuals who have self‐harmed ‘do not deserve to come to ICU’ (5.4%, *n* = 2/37).	4.1. Is the sampling strategy relevant to address the research question? Yes 4.2. Is the sample representative of the target population? ICU nurses working on a large inner‐city unit. 4.3. Are the measurements appropriate? Yes 4.4. Is the risk of nonresponse bias low? 40/124 respondents 4.5. Is the statistical analysis appropriate to answer the research question? Yes	MH patients are violent and aggressive. ‘They’ don't belong in ICU. ‘Those types of patients’. ‘They’ need someone with special skills.

Abbreviations: ICU, intensive care unit; MH, mental health; MMAT, Mixed Methods Appraisal Tool.

Deep iterative reading followed, with the aim of identifying patterns and themes.^21^ Integrative reviews commonly use a thematic approach to data analysis.^15^, ^16^ Thematic analysis is flexible, and facilitates the creation of a rich and complex account of the analysed data.^21^ The included texts were initially coded (AT). For example, labels were identified as a code and extracts were recorded, such as ‘the self‐harmers’. The codes were then reviewed and collated into themes (AT), which were agreed by both authors (AT and JB). The text coded as ‘labels’ contributed to the theme ‘Those types of patients’. Finally, themes and extracted data were checked back against the original text to ensure that decontextualization did not occur.

Papers were appraised using the Mixed Methods Appraisal Tool (MMAT).^22^ The tool includes two general screening questions for all types of studies, then separate sets of prompts for qualitative studies and different quantitative methods (Hong et al., 2018). All included studies were found to meet the tool's first basic screening question as they had a clear aim. All studies except the two discursive papers^23^, ^24^ met the second question because the collected data allowed the authors to address their research question. The two discursive papers combined expert opinion regarding case studies with published research and, as such, collected no empirical data.

The MMAT produces a descriptive summary of appraisal. Summary scores can be calculated, but are not recommended by the authors of the tool because of the risk of masking flaws within different aspects of a paper.^22^ No studies were excluded from the review on grounds of quality; however, appraisal facilitated the identification of several limitations in the included studies. A summary of the MMAT results is included in Table 2.

### 2.4 Findings

Of the 620 papers identified, eight studies were selected for inclusion. There was a paucity of high‐quality empirical evidence relating to the focus of the review. This necessitated the inclusion of lower‐quality studies. The included studies comprised four qualitative, two quantitative and two discursive papers based on case studies (Figure 1, ^17^). Most studies originated from Australia (*n* = 5), followed by New Zealand (*n* = 1), United States (*n* = 1) and Turkey (*n* = 1). Two discursive papers focused on case studies and relevant discussion by the authors. One^24^ focused on a single case, whilst the other^23^ offered two fictitious cases for discussion. As such, neither involved formal data collection, limiting their quality and the transferability of their findings. Both papers were included in the review despite this because they offered insight into how patients with an MH disorder should be ideally managed in ICU, and how critical physical illness and mental illness can be interrelated.^23^, ^24^ The remaining papers all took place in single centres, potentially limiting generalizability and transferability of the results.^8^, ^25^, ^26^, ^27^, ^28^, ^29^ Sample sizes were generally small across all the included studies (*n* = 15–40), and five of the eight included studies were undertaken in Australia, with none being based in the United Kingdom or Europe, further limiting transferability. In addition, the two papers by Corfee et al. (2019 and 2020) represented different analyses of the same dataset. The perception of MH health disorders is a potentially emotive topic. There is a risk that, in interviews, participants may underreport their prejudices to meet perceived social and professional standards (Table 3).

**TABLE 3:** Themes summary.

Theme	Summary	Example data	Contributing papers
‘Those types of patient’	This theme explored the notion of ‘othering’ when ICU nurses discuss patients with a MH disorder ‘Othering’ can be achieved through language (‘those types of patient’) and establishes a social difference which can impact on the quality of care delivered to the marginalized group	Patients with MH disorders were described variously as ‘the self‐harmers’, ‘the suicide attempts’, ‘mental health patients’, ‘dangerous patients’ (Patterson et al., 2023) and ‘these people’ (Corfee et al., 2019) We're all guilty of stigmatizing mental health patients. It is hard after so much compassion fatigue, to not see schizophrenia and roll your eyes (Patterson et al., 2023).	Corfee et al., 2019; Corfee et al., 2020; Flaws et al., 2023; Murch, 2016; Patterson et al., 2023; Weare et al., 2019
Patients with mental health disorders are all violent and aggressive	This theme explored the assumption that patients with a MH disorder were more likely to be violent towards nursing staff. This was shown to influence how care was delivered. For example, nurses were keen to have easy access to restraints.	Patients with a MH disorder were thought to be dangerous assumed to ‘cause problems’ and, in contrast to ICU patients without a co‐morbid MH disorder, be culpable for any aggressive behaviour (Patterson et al., 2023) MH patients need ‘double safety’ compared with other patients. This is achieved through the removal of devices which could be potentially used as weapons and having chemical and physical restraints within easy reach (Corfee et al., 2020).	Aktaş & Arabacı, 2023; Corfee et al., 2019; Corfee et al., 2020; Flaws et al., 2023; Murch, 2016; Patterson et al., 2023; Weare et al., 2019
‘They’ don't belong in ICU	This theme explored views held by ICU nurses about whether it was appropriate to admit a patient with a MH disorder to ICU or, in the case of patients who had attempted suicide, whether they were ‘deserving’ of ICU‐level care	Patients with MH disorders were described as having ‘*complex emotional baggage and problems*’ (Patterson et al., 2023) which were at the ‘*opposite end of the spectrum*’ and ‘*out of scope*’ for ICU‐based care (Patterson et al., 2023) I don't even know why we keep trying to bring her back. People should just let her go. She should just do a good job; it's really not that hard to kill yourself. She's taking up bed space (Corfee et al., 2019).	Corfee et al., 2019; Corfee et al., 2020; Flaws et al., 2023; Murch, 2016; Patterson et al., 2023; Weare et al., 2019
‘They’ need someone with special skills	This theme explored the notion that patients with a MH disorder could not be fully cared for by an ICU nurse. This could be because of lack of perceived ‘special’ skills, or the belief that ‘someone else’ should be responsible for MH patients.	Nurses felt inadequately prepared and skilled to deal with patents with a MH disorder (Bone & Smith, 2012). They described a fear of ‘saying the wrong thing’ (Weare et al., 2019) and avoided patients with a MH disorder if possible. A need for training and consistent support was noted.	Aktaş & Arabacı, 2023; Bone & Smith, 2012; Corfee et al., 2020; Flaws et al., 2023; Murch, 2016; Patterson et al., 2023; Weare et al., 2019

Abbreviations: ICU, intensive care unit; MH, mental health.

Four themes were identified: (1) ‘Those types of patient’, (2) Patients with MH disorders are all violent and aggressive, (3) ‘They’ don't belong in ICU and (4) ‘They’ need someone with special skills.

#### 2.4.1 ‘Those types of patients’

The first theme explored the notion of ‘othering’ when speaking about patients with MH diagnoses. Six studies contributed to this theme.^8^, ^23^, ^26^, ^27^, ^28^, ^29^ Othering refers to the rendering of one group, in this case patients with a MH disorder, into one homogenous mass (‘the other’), then placing them in opposition to another privileged group who hold professional and social power (‘the one’, in this case, ICU staff). The homogenous mass lacks any nuance, difference or humanity (heterogeneity) and can thus be reduced to character traits based on assumptions. Social difference is therefore established and reproduced through methods such as the language used to subjectively describe patients with a MH diagnosis.^8^

Patients with MH disorders were described variously as ‘the self‐harmers’, ‘the suicide attempts’, ‘mental health patients’, ‘dangerous patients’^28^ and ‘these people’.^8^ Such descriptors serve to separate them from ‘average patients’, or those who did not have a co‐morbid MH disorder. Reducing a patient group to a homogenous and stereotyped typification can precede discriminatory practices against a group. A participant in the interviews undertaken by Corfee, Cox and Windsor^8^ noted that staff were more likely to initiate chemical restraint with patients with a MH disorder. The generation of typification based in stigma also led nursing staff to engage in behaviours such as close observation and ostracization.^26^ Such actions were not based on an objective assessment of risk, but rather on the possibility of what a patient ‘might’ do, based on accepted social knowledge of the group.^26^

Survey data collected by Weare et al.^29^ from 40 inner‐city ICU nurses suggested that the majority (84.2%, *n* = 32) believed that understanding their patients' psychological state is important for optimal ICU treatment. However, 29.7% (*n* = 11) believed that MH patients were not treated with empathy in their unit. 60% (*n* = 24) of ICU nurses surveyed by Murch^27^ suspected that negativity was demonstrated towards patients with an MH disorder in their ICU. Perceived rejection or devaluation was linked by Flaws, Patterson^23^ to behaviour which did not fit into the ICU norm, such as physical agitation or ‘acting out’.^28^ A participant in the interviews undertaken by Patterson, Flaws^28^ commented that:

We're all guilty of stigmatising mental health patients. It's hard after so much compassion fatigue, to not see schizophrenia and roll your eyes.

24.3% (*n* = 9) of participants in the study undertaken by Weare et al.^29^ experienced frustration and irritation when caring for patients admitted to ICU because of deliberate self‐harm. Corfee, Cox and Windsor^8^ noted that nurses appeared to regard MH patients as ‘*ineffectual stewards of their own health concerns’* and blamed this patient group for their admissions

ICU patients with co‐morbid MH disorders were noted by participants from two studies to behave in ways that further differentiated them from ‘typical’ ICU patients. 62.2% (*n* = 23) of the respondents to Weare et al.'s (2019) survey believed MH patients behaved in an unpredictable fashion. In contrast, Flaws, Patterson^23^ noted that such behaviour might be a form of coping mechanism which has previously been helpful to the person. However, behaviour such as walking around the unit or being unwilling to lie still may be considered counterproductive and unsafe:

…that makes it dangerous at nighttime for them to walk around. We don't like that, so we give them medication to force them to stay in their bed space.^8^

Here, behaviour which is seen as outside the norm carries the penalty of chemical restraint. This action is justified as necessary for the maintenance of the safety of other patients. A further participant described constructing a physical ‘barricade’ to prevent patients from leaving their bed area.^26^ The subjective ‘*we don't like that’* ^8^ suggests a lack of willingness to adapt care.

#### 2.4.2 Patients with MH disorders are not all violent and aggressive

The second theme explored how assumptions about patients with an MH disorder being more likely to behave in a violent manner can impact the care they receive from ICU nurses. Violence in clinical workplaces is increasing and is likely to be under‐reported. Patterson, Flaws^28^ note that violence and aggression are more likely in critical care areas. However, assumptions about patients with an MH disorder being more likely to be aggressive can reduce the quality of care they receive. Six studies contributed to this theme.^8^, ^23^, ^25^, ^26^, ^27^, ^28^, ^29^

Patterson, Flaws^28^ interviewed 19 ICU nurses and found that they associated ‘dangerousness’ with MH patients and believed that they were more likely to exhibit violent and aggressive behaviours. Such patients were assumed to ‘cause problems’ and, in contrast to ICU patients without a co‐morbid MH disorder, to be culpable for any aggressive behaviour.^28^ Most respondents (73.7%, *n* = 29/40) reported nervousness when allocated to care for a patient with a MH disorder,^29^ and others worried for their personal safety.^27^ One nurse interviewed by Patterson, Flaws^28^ stated that they believed ‘acting out’ was a normal approach to life or ‘these patients’. Nurses also described how they found it ‘confronting’ and ‘difficult’ to engage with patients who were experiencing hallucinations or delusions.^25^, ^29^

Such concerns are amplified through the stereotyping of behaviours. The assumption that all patients with a MH disorder are violent and aggressive led some nurses to peremptorily act to maintain the safety of themselves and their patients. A participant in the interviews held by Corfee, Cox and Windsor^26^ explained how MH patients need ‘double safety’ compared with other patients. This is achieved through the removal of devices that could be potentially used as weapons and having chemical and physical restraints within easy reach.^26^ It was also noted that a nurse's threshold for reacting to distressed or irritable behaviours was lower when managing a patient with a MH disorder.^8^

Nurses also acted on socially reproduced concerns about the potential for MH patients harming themselves whilst admitted to ICU. One participant noted that, if a patient was allowed to shower independently, they could use the opportunity to harm themselves because ‘*they do stuff like that*’.^26^ Other patients were permitted to shower alone because ‘*they don't do that sort of thing*’.^26^

#### 2.4.3 ‘They’ do not belong in ICU

This theme explored views held by ICU nurses about whether it was appropriate to admit a patient with a MH disorder to ICU. Some papers explored how nurses saw patients who self‐harm as undeserving of ICU care,^8^, ^23^, ^29^ whilst others commented on how the ICU environment was inappropriate for patients with a MH disorder.^27^, ^28^ Five papers contributed to this theme.

59.4% (*n* = 27) of the respondents to the survey issued by Weare et al.^29^ did not feel that ICU was an appropriate environment in which to nurse patients with MH disorders. The challenge of developing a therapeutic rapport between nurse and patient was linked to the physical ICU environment,^27^ with nurses commenting that they also lacked time to fully engage with the needs of a patient with a MH disorder.^29^ The ICU environment was described as a ‘*foreign place’* where patients would, understandably, not feel safe when they awoke from sedation to find themselves physically restrained.^28^ This would lead, in this participant's experience, to a ‘fight or flight’ response to attempt to ‘*get safe*’.^28^ Patients with MH disorders were described as having ‘*complex emotional baggage and problems*’^28^ which were at the ‘*opposite end of the spectrum*’ and ‘*out of scope*’ for ICU‐based care.^28^ The presence of MH patients in ICU was also deemed to disturb other patients and nursing routines.^28^, ^29^

A participant in the interviews undertaken by Corfee, Cox and Windsor^8^ stated that ICU was ‘*in the business of saving lives*’. A socially constructed image of ICU is placed in opposition to the stereotyped homogeny of MH patients. A minority of nurses (5.4%, *n* = 2) surveyed by Weare et al.^29^ believed that patients who had deliberately self‐harmed did not ICU care, whilst 37% (*n* = 11) of the respondents to the survey undertaken by Murch^27^ found it hard to sympathize with patients who were recurrently admitted following deliberate self‐harm. Dehumanization and disempowerment were demonstrated towards a patient who had experienced several ICU admissions following deliberate self‐harm:

I don't even know why we keep trying to bring her back. People should just let her go. She should just do a good job; it's really not that hard to kill yourself. She's taking up bed space.^8^

It is important to note that such views were expressed by a small minority. However, Flaws, Patterson^23^ noted that such opinions can cause guilt to the nurse themselves, as well as impacting negatively on the care given to the patient. The judgement regarding the way this patient has chosen to live her life is incongruent with the professional identity of a nurse^23^ and may lead to feelings of guilt or inadequacy.

#### 2.4.4 ‘They’ need someone with special skills

This theme explored the notion that patients with a MH disorder could not be fully cared for by an ICU nurse. This could be because of a lack of perceived ‘special’ skills, or the belief that ‘someone else’ should be responsible for MH patients. Seven studies contributed to this theme.^23^, ^24^, ^25^, ^26^, ^27^, ^28^, ^29^

Nurses felt inadequately prepared and skilled to deal with patents with a MH disorder^24^ and wanted further educational support.^27^ 100% of the respondents to the Weare et al.^29^ survey felt that they could be better prepared to manage MH disorders. They expressed a fear of saying ‘*the wrong thing*’ (48.7%, *n* = 19)^29^ and a reluctance to approach the patient in case they caused them to become upset.^26^ This was described as leading to a culture of surveillance and distance.^26^ This is in direct contrast to the trauma‐informed compassionate care advocated for by Flaws, Patterson.^23^ One participant commented that often ICU nurses ‘*don't want to talk deeper*’,^28^ suggesting a culture of unwillingness to engage.

There appeared to be a consensus that patients with MH disorders needed someone with specialized training to be allocated to care for them. A participant in the Patterson, Flaws^28^ study commented that MH and ICU nurses have very different skillsets and that ‘*no‐one feels equipped to manage a patient who sits in both spaces*’. Bone and Smith^24^ noted that MH is often ‘lost’ in ICU and that the physical illness is prioritized. However, despite this, the MH illness becomes a way of labelling the patient, in a way that is not seen with a physical illness.^24^ 86.5% (*n* = 32) of the ICU nurses surveyed by Weare et al.^29^ believed they could provide better care if they were supported by a trained MH nurse. Participants in the interviews held by Aktaş and Arabacı^25^ stated variously the need for a psychiatrist on ICU, someone with training whose job it was to care of patients with MH disorders, or a special unit in the hospital. They felt that the hospital management did not see how they were struggling to manage MH patients on ICU.

## 3 DISCUSSION

This integrative review provides an overview of how ICU nurses perceive patients with a co‐morbid MH disorder who are admitted to ICU. This topic appears to be under‐researched and, as a result, includes evidence which is limited in terms of quality and transferability. This review brings together a synthesis of eight published studies. Four themes were identified: (1) ‘Those types of patient’, (2) Patients with MH disorders are violent and aggressive, (3) ‘They’ don't belong in ICU and (4) ‘They’ need someone with special skills. The themes highlighted the stigma experienced by patients with MH disorders. They also explored some common misconceptions about this patient group, and how these might impact on the quality of the care provided. Finally, a need for support and education and support for ICU nurses caring for MH patients was highlighted.

A culture of ‘othering’ MH patients was demonstrated in several studies.^8^, ^26^, ^28^ In ‘othering’ a person, or group, is differentiated from, and thus excluded and subordinated by, a dominant social group.^30^ This can be observed in nursing and health care on several levels. Broadly, patients with MH disorders experience marginalization and perceived exclusion from the health care system as a whole^2^ because of their potential need to deviate from standardized care pathways.^9^ Jacob et al.^9^ suggest a concept of ‘structural othering’, where a person with a stigmatized condition is subject to discrimination on a macro level, through poor provision and funding of supportive services. People with MH disorders are more likely to die from preventable causes and suffer with long‐term illnesses.^2^, ^31^ In recent years, it has been demonstrated that persons with MH disorders are more likely to be hospitalized from or die from COVID‐19.^32^ Increased hospitalization rates and support for long‐term conditions have considerable cost implications to health care providers.^31^

The reasoning behind this is complex. Patients with MH disorders experience barriers to accessing health care^2^, ^8^, ^23^, ^31^ and are also more likely to engage in behaviour that risks their health, such as smoking.^33^ In ICU, there appears to be a belief that patients with MH disorders, especially those who engage in deliberate self‐harm or risky behaviour, do not deserve intensive care.^26^ However, there is a need to generate further research evidence to fully explore how this belief impacts patient care.

MH patients, like those with hyperactive delirium, do not conform to the ‘norm’ in ICU.^30^, ^34^ Their behaviour, such as needing to get up and walk around,^23^ is disruptive to ICU nursing routines, often rendering them unpopular.^34^ Critical care nurses have been noted to engage heavily with ritual and routine. Behaviour which defies or disrupts these routines can be perceived as a challenge to the nurses' power.^35^, ^36^ In clinical practice, difference can lead to a patient being seen as ‘difficult’ or ‘challenging’.^9^ Constraints, such as poor staffing, inflexibility and lack of resources, can lead nurses to systematically exclude ‘challenging’ patients from ‘normal’ care.^9^ This review has identified a number of ways that ICU nurses demonstrate their power, from restricting a patient's access to equipment or a private shower,^8^, ^26^ to affording blame and culpability for aggressive behaviour whilst ‘normal’ patients were excused.^28^ However, there are also other, more subtle, ways that nurses can demonstrate their power. Browne^37^ undertook an ethnographic study which aimed to explore how Canadian nurses act towards female patients of First Nations descent. In her paper, she identified clear binaries between ‘them’ and ‘us’ in terms of behaviour and communication. A First Nations woman was told she could use the ward phone on one occasion only, but a white man was not subject to this rule.^37^ This was based on the belief that, for First Nations people, if you ‘give an inch, they will take a mile’. Similarly, this review demonstrated discriminatory behaviour based on the inaccurate belief that patients with a MH disorder are likely to be violent. In reality, they are more likely to be victims of violence.^23^ The judgements that nurses may pass on the decisions such patients make and their behaviour lack congruence with their identity as a nurse. This can lead to feelings of guilt^23^ and compassion fatigue.^38^

Exclusion of patients can be a result of ‘othering’. This can be an intentional or unintentional act. Unintentional exclusion can indicate limited self‐knowledge on the part of the nurse.^30^ Exclusion creates distance between the nurse and patient and limits their therapeutic engagement.^30^ Flaws and Patterson^23^ discuss how a compassionate trauma‐informed approach is necessary to deliver holistic care to patients with a MH disorder in ICU. However, the results of this review suggested a lack of knowledge and perceived lack of skills amongst ICU nurses when managing MH. Nurses who feel that they lack the skills to manage a certain patient may choose to avoid interactions with that patient.^35^ Respondents surveyed by Weare et al.^29^ and Murch^27^ expressed a need for further education. Educational programmes in both A&E and perinatal health^39^, ^40^ have demonstrated some positive impact in terms of reduced urgent admissions to inpatient care and self‐reported improved attitudes towards patients with MH disorders. However, as noted by Patterson and Flaws,^28^ increased knowledge alone is not enough. The stigma against patients with MH is well established, as demonstrated by this review. Misconceptions are rife, and the language used to describe the patient group reinforces their place as the ‘other’. To deliver holistic, patient‐centred and trauma‐informed care, nurses must be supported. Sharda, Baker and Cahill^41^ interviewed patients with a personality disorder who were admitted to general hospital wards. They found that the separation of mental and physical health care caused patient distress and disruption to their treatment and called for better integration between the services. Thornicroft and Sunkel^42^ identified the value of persons with lived experience of MH disorders in the co‐production of anti‐stigma programmes. ICU practice might be similarly improved, and bias reduced, through the use of patient voices in staff education.

There has been a global drive towards the equal treatment of mental and physical health conditions.^43^, ^44^ However, professional bodies have called for improvements in the management of patients with a MH disorder in general hospital settings.^45^ However, this review suggests that MH care is still seen as separate from the care delivered in ICU. Participants in one study called for greater support from MH trained nurses^25^; however, this would place a further burden on the already stretched MH services.^41^ In the United Kingdom, liaison MH services exist within some general hospitals. This multi‐disciplinary service offers assessment and care planning support for patients with MH disorders admitted to general hospitals. However, these services require a referral from health care staff, and participants in the Sharda, Baker and Cahill^41^ study note that they were insufficiently integrated into care and challenging to access and lacked the resources to provide holistic support. The results of this review suggest that ICU nurses would welcome support from specialized practitioners and that mental and physical health services need to be better integrated.

### 3.1 Strengths and limitations

This review has drawn upon a range of evidence to explore and understand how ICU nurses perceive patients with a co‐morbid MH disorder. The search and synthesis were undertaken using established methods,^16^ and appraisal was guided by a validated tool.^22^ The review has identified several areas of practice that require further research.

This review was limited by the paucity of evidence on this topic. The search returned only eight studies, all of which were included in the review. The majority of the studies (*n* = 6) were based in single centres with small sample sizes (*n* = 15–40), and two were discursive papers that considered case studies rather than empirical evidence. No studies originated from Europe. This is a further limitation to this review, as it potentially inflates the impact of cultural MH biases. For example, five papers originate from Australia. There is increasing evidence linking racism against the indigenous population with poor physical and MH care.^46^ As such, this review may not present a balanced perspective on how ICU nurses perceive patients with MH disorders.

### 3.2 Recommendations for further research

This review forms the initial step of a research project which aims to explore how UK‐based ICU nurses perceive patients with a co‐morbid MH disorder. This review found no empirical data from the United Kingdom, and the authors consider that it is important to study this topic in the context of the United Kingdom to facilitate targeted recommendations for practice, education and support in clinical areas. Focus groups are planned with the aim of exploring how nurses share information about MH disorders and construct identities for their patients. This review suggests that this practice leads to the perpetuation of stigma and negatively affects care delivery.

## 4 CONCLUSION

To conclude, this integrative review has identified a paucity of high‐quality evidence regarding how patients with a MH disorder are perceived by ICU nurses. However, the findings do suggest that stigma, misconceptions and the continued separation of physical and mental health care could have a detrimental impact on the care of ICU patients with a co‐morbid MH disorder. Further research is indicated to explore this topic in a UK setting and to identify how educational support and multi‐disciplinary collaboration could reduce bias against MH patients and optimize the care they receive in ICU.

## Data Availability

Data sharing is not applicable to this article as no new data were created or analyzed in this study.
